# Comprehensive characterization of cardiac morphology and function in adult patients with phenylketonuria using CMR

**DOI:** 10.1186/1532-429X-16-S1-P246

**Published:** 2014-01-16

**Authors:** Jan-Hendrik Hassel, Nikolaus Tilling, Lenka Bosanska, Bernhard Schnackenburg, Daniel Messroghli, Alexander Berger, Rolf Gebker, Christopher Schneeweis, Eckart Fleck, Ursula Plöckinger, Sebastian Kelle

**Affiliations:** 1 Cardiology, German Heart Institute Berlin, Berlin, Germany; 2 Interdisziplinäres Stoffwechsel-Centrum, Charité-Universitätsmedizin Berlin, Campus Virchow-Klinikum, Berlin, Germany; 3 Philips Healthcare Systems, Hamburg, Germany

## Background

Phenylketonuria (PKU) is one of the most common inherited metabolic disorders. The molecular pathway of neurological damage is not yet sufficiently understood. To date, there is a lack information about cardiac involvement related to the disease. This study aims to characterize cardiac morphology and function in adult patients with PKU using cardiovascular magnetic resonance (CMR).

## Methods

28 patients with PKU (age 30 ± 9 years/mean ± SD) underwent a comprehensive CMR protocol at a 1.5T CMR scanner (Philips, Achieva) including assessment of left ventricular (LV) volume and mass. In addition, T1 measurements pre- and post-administration of gadolinium for evaluation of extra corpuscular volume (ECV) and tagging for quantitative analysis of left ventricular circumferential strain (Ecc) were performed. 8 healthy age-matched volunteers underwent a similar protocol and served as controls for the ECV values. LV parameters and Ecc were compared to reference values from previous studies with similar data setup [1-3].

## Results

**Figure 1:**
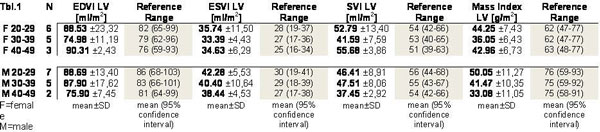
**left ventricular mass index (LVMI) in patients with phenylketonuria separated after age and sex**.

**Figure 2:**
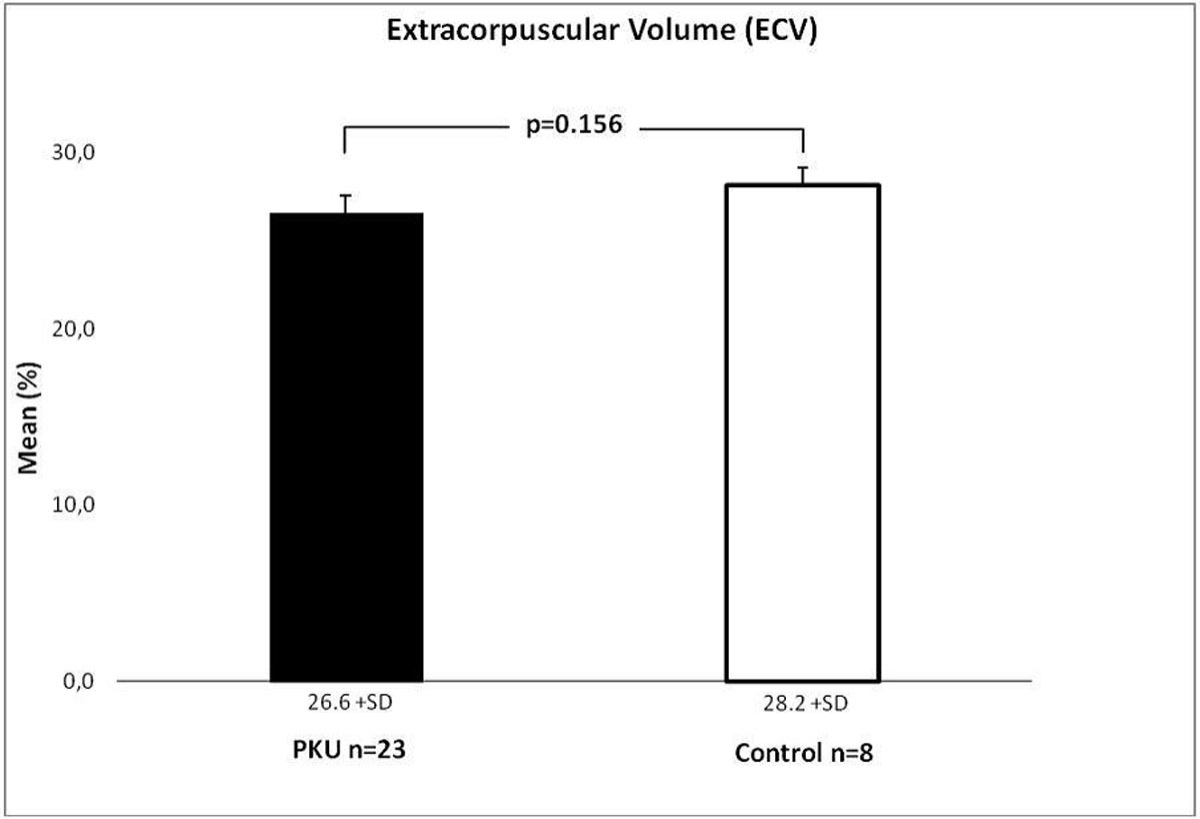
**ECV in patients with phenylketonuria and healthy controls**.

CMR exams were successfully performed in all patients. As shown in Figure 1 LV mass index was reduced to the lower 95% confidence interval of the reference values [4,5] in each subgroup. ECV showed no significant difference between PKU patients (0.27 ± 0.03/mean ± SD) and the control group (0.28 ± 0.02/mean ± SD) (Figure 2) p = 0.15. PKU patients had higher Ecc values (= -0.22%) compared to reference values (Ecc = -0.20%) with similar segmental patterns.

## Conclusions

The results of this study indicate that in PKU patients compared to healthy controls LV mass indexed to BSA is reduced to the reference values; we found increased average Ecc parameters and normal ECV values. Further investigations in larger patient groups and older PKU patients are necessary for evaluation of cardiac involvement of the disease over time and its consequences.

## Funding

None.
